# Integrative Transcriptomic and Machine Learning Analysis Identifies Key Senescence-Associated Secretory Phenotype Genes Associated With Immune Dysregulation in Periodontitis

**DOI:** 10.1155/humu/3637248

**Published:** 2025-12-02

**Authors:** Jing Zeng, Jing Huang, Juan He, Jianqin Tan, Mianmian Duan, Yanyan Song, Lin Yang

**Affiliations:** ^1^Department of Stomatology, The Second Affiliated Hospital of Guizhou University of Traditional Chinese Medicine, Guiyang City, Guizhou Province, China; ^2^Department of Stomatology, The Central Hospital of Enshi Tujia and Miao Autonomous Prefecture, Enshi City, Enshi Tujia and Miao Autonomous Prefecture, Hubei Province, China; ^3^Department of Stomatology, Guiyang Stomatological Hospital, Guiyang City, Guizhou Province, China

**Keywords:** biomarkers, immune infiltration, machine learning, multiomics, periodontitis, senescence-associated secretory phenotype, transcriptomics

## Abstract

Periodontitis (PD) is a chronic inflammatory disorder marked by immune dysregulation and progressive tissue destruction. Cellular senescence and the senescence-associated secretory phenotype (SASP) have been increasingly recognized as pivotal drivers of chronic inflammation. However, their specific contributions to PD remain insufficiently clarified. In this study, integrative bioinformatic analyses were conducted across transcriptomic datasets, employing least absolute shrinkage and selection operator, support vector machine–recursive feature elimination, and eXtreme gradient boosting algorithms to identify SASP-related genes of significance. ICAM1, CXCL12, and MMP3 were found to be markedly upregulated in PD and demonstrated strong diagnostic potential through receiver operating characteristic and artificial neural network models. Functional enrichment analysis indicated their involvement in immune cell adhesion, migration, and infection-associated pathways. Immune infiltration profiling revealed disrupted immune landscapes, with ICAM1 exhibiting a negative correlation with resting mast cells. Experimental validation using real-time quantitative polymerase chain reaction and immunohistochemistry on clinical samples confirmed elevated expression of these genes at both the mRNA and protein levels. Moreover, dexamethasone was identified via molecular docking as a potential therapeutic compound targeting ICAM1 and CXCL12. Collectively, these findings advance the understanding of SASP associated with immune regulation in PD and suggest potential biomarkers and therapeutic targets for early diagnosis and intervention.

## 1. Introduction

Periodontitis (PD) is a chronic, destructive inflammatory disorder affecting the gums and periodontal tissues [1]. Its principal pathological features include periodontal pocket formation, inflammation of pocket walls, alveolar bone resorption, and progressive loosening of teeth. The inflammatory process involves not only the gingival soft tissues but also deeper supporting structures, including the periodontal ligament, alveolar bone, and cementum. Moreover, PD can elicit systemic inflammatory and immune responses through microorganisms and their products present in the subgingival plaque biofilm [2, 3]. Consequently, early diagnosis and timely intervention in PD are indispensable for preventing disease progression and mitigating systemic inflammation and immune activation. Currently, clinical management primarily emphasizes elimination of etiological factors, suppression of pathogenic plaque, and pharmacological modulation of the disease [4]. Nevertheless, owing to the multifactorial complexity of PD, therapeutic outcomes remain inconsistent, and effective treatment is not universally achieved [5]. Furthermore, because early manifestations of PD are frequently subtle, delayed recognition often results in disease advancement [6]. Therefore, elucidating the molecular mechanisms underlying PD and identifying potential molecular targets are essential for improving early diagnostic strategies and advancing personalized therapeutic approaches.

The senescence-associated secretory phenotype (SASP) denotes a repertoire of proinflammatory cytokines, chemokines, and matrix metalloproteinases secreted by senescent cells, constituting a defining feature of cellular senescence (L. [7]). SASP exerts pivotal roles in diverse physiological and pathological contexts, including stimulation of nonsenescent cell proliferation, modulation of the cellular microenvironment, and involvement in chronic inflammation and immune responses (X. [8]). Specifically, SASP encompasses proinflammatory cytokines (e.g., IL-1*α*, IL-1*β*, IL-6, and IL-8), growth factors (e.g., HGF, TGF-*β*, and GM-CSF), chemokines (e.g., CXCL-1/3 and CXCL-10), and matrix metalloproteinases (e.g., MMPs) [9]. In the pathogenesis and progression of PD, SASP not only alters the local microenvironment but may also intensify inflammatory responses via systemic immune modulation (Q. [10]). Recent studies have further elucidated the specific roles of the SASP in PD. Evidence has demonstrated that senescent cells, through interactions with bacteria, release SASP factors that reprogram the microenvironment, inducing paracrine signaling in adjacent cells, thereby exacerbating chronic periodontal inflammation and contributing to further alveolar bone resorption [11]. Other studies have shown that in inflamed gingiva, human gingival fibroblasts acquire a senescent phenotype due to oxidative stress–induced DNA and mitochondrial damage, which in turn activates neutrophils and macrophages through the secretion of SASP factors (S. [12]). Furthermore, some researchers have proposed that the colony-stimulating factor 1 receptor (CSF1R) inhibitor PLX3397 can reduce macrophage senescence via the PI3K/AKT/FOXO1 signaling pathway, thereby alleviating experimental PD [13]. Additionally, clinical investigations have reported a marked age-dependent increase in both prevalence and severity of PD (S. [14]). Although the roles of SASP in PD have gradually been recognized, which SASP genes hold the greatest diagnostic or therapeutic value remains unclear. Therefore, the identification of senescence-associated secretory phenotype-related genes (SASPRGs) in PD is essential for elucidating its molecular mechanisms, establishing potential diagnostic biomarkers, and advancing therapeutic target discovery.

In this study, transcriptomic data from public databases were utilized, and differential expression analysis and protein–protein interaction (PPI) network screening, as well as machine learning approaches, were applied to identify key genes associated with SASP in PD. Through integration of expression validation, receiver operating characteristic (ROC) curve analysis, and artificial neural network (ANN) modeling, the functions and potential mechanisms of these genes in PD were further investigated. These findings establish a theoretical foundation for molecular mechanism studies of PD and contribute to the advancement of precision treatment.

## 2. Materials and Methods

### 2.1. Data Collection

Gene expression data for the GSE10334 and GSE223924 datasets associated with PD were retrieved from the Gene Expression Omnibus database. Specifically, the GSE10334 dataset (training set) (platform: GPL570) comprised 183 PD patient samples and 64 healthy gingival tissue controls (H. [15]). The GSE223924 dataset (validation set) (platform: GPL24676) included 10 PD patient samples and 10 healthy peripheral gingival tissue controls [16]. Normalization of the GSE10334 dataset was performed by first determining whether the data required a log2 transformation, followed by conventional array normalization at the probe level. After that, the probes were mapped to genes, and duplicates were removed based on average expression, retaining only protein-coding genes. For the GSE223924 dataset, the prenormalized FPKM matrix provided by the GEO database was directly used. Additionally, 81 SASPRGs were obtained from one study [17], and 83 SASPRGs were obtained from another [18]. After data integration and removal of duplicates, 153 SASPRGs were identified in total (Table S1).

### 2.2. Differential Expression Analysis

To identify differentially expressed genes (DEGs) between PD and control samples in the GSE10334 dataset, differential expression analysis was conducted using the limma package (v 3.58.1) [19] under the thresholds of *p* adjust < 0.05 and |log2FoldChange (FC)| > 0.5 (D.-B. [20]). Visualization of DEGs was performed with volcano and heat map plots generated by the ggplot2 package (v 3.4.1) [21].

### 2.3. Identification of Candidate Genes

Intersections between DEGs and SASPRGs (candidate genes) were analyzed using the ggvenn package (v 0.1.10) [22]. Gene Ontology (GO) enrichment analysis was employed to functionally annotate genes based on the GO database, encompassing three categories: biological process (BP), cellular component (CC), and molecular function (MF). Kyoto Encyclopedia of Genes and Genomes (KEGG) pathway enrichment analysis was performed using the KEGG database to annotate pathways of all identified protein sets or screened differentially expressed proteins, thereby determining the principal metabolic and signal transduction pathways associated with these genes or proteins. GO and KEGG enrichment analyses of candidate genes were conducted with the clusterProfiler package (v 4.8.3) (T. [23]) (*p* < 0.05), and the results were visualized using ggplot2 (v 3.4.1). Candidate genes were subsequently submitted to the STRING database to construct a PPI network (confidence score ≥ 0.4)[24], and the network was visualized using Cytoscape software (v 3.10.2) (P. [25]). Furthermore, the CytoHubba plugin of Cytoscape software (v 3.10.2) was applied with the top five algorithms, maximal clique centrality (MCC), maximum neighborhood component (MNC), density of maximum neighborhood component (DMNC), degree, and edge percolated component (EPC), to evaluate the significance of each candidate gene. The intersection of the Top 15 proteins derived from each algorithm was designated as the final set of candidate genes for subsequent analysis.

### 2.4. Identification of Key Genes

Machine learning, a widely applied analytical approach for gene feature screening, was employed to refine the final candidate genes obtained from the PPI analysis. Three algorithms, least absolute shrinkage and selection operator (LASSO) regression, support vector machine–recursive feature elimination (SVM-RFE), and eXtreme gradient boosting (XGBoost), were applied. LASSO regression (glmnet package, v 4.1-8) (Y. [26]) constructs a penalty function that reduces the coefficients of less informative variables to zero, thereby excluding them from the model. And a 10-fold cross-validation was conducted. Genes exerting greater influence on PD were retained as LASSO genes, and the corresponding regression coefficients were recorded. Based on all samples in the GSE10334 dataset, SVM-RFE (caret package, v 6.0-94) (Z. [27]) was conducted with 10-fold cross-validation to rank the importance of each final candidate gene. Error rates and accuracies of iterative combinations were assessed, and the combination with the highest accuracy was designated as optimal. Genes selected at this stage were defined as SVM-RFE genes. Subsequently, XGBoost (xgboost package, v 2.0.3.1) [28] was applied to the final candidate genes, and genes with high importance scores were identified as XGBoost genes. Finally, the ggvenn package (v 0.1.0) [22] was used to compute the intersection of LASSO, SVM-RFE, and XGBoost genes, with the overlapping genes designated as characteristic genes [29].

To assess the expression of characteristic genes and identify key genes in the GSE10334 and GSE223924 datasets, the Wilcoxon test was performed to evaluate expression differences between PD and control samples (*p* < 0.05). Results were visualized with boxplots generated using the ggplot2 package (v 3.4.1) (Q. [30]). Genes consistently exhibiting significant differential expression across both datasets were shortlisted as candidate key genes.

To determine the discriminatory capacity of candidate key genes, ROC curve analysis was conducted using the pROC package (v 1.18.5) [31] across all samples in both datasets. The area under the curve (AUC) was applied to measure predictive sensitivity and specificity, with values closer to 1 indicating superior accuracy. Genes with AUC > 0.7 in both datasets were defined as key genes.

### 2.5. Construction of an ANN

An ANN is a machine learning model designed to emulate the learning processes of biological neural networks. To assess the predictive capability of key genes for PD, an ANN model was constructed using the neuralnet package (v 1.44.2) (Y. [32]) with all samples from the GSE10334 dataset. A 10-fold hierarchical cross-validation was implemented when constructing the ANN model. Subsequently, the pROC package (v 1.18.5) [31] was applied to perform ROC curve analysis, and an AUC > 0.7 was considered indicative of favorable predictive performance. In addition, the ANN model was validated in the validation set GSE223924 using the same method.

### 2.6. Gene Set Enrichment Analysis (GSEA)

To elucidate the biological functions and pathways associated with key genes in PD, based on all samples from the GSE10334 dataset, the c2.cp.kegg.v7.4.symbols.gmt (v 7.4) file from MSigDB was used as the reference gene set. The psych package (v 2.4.3) [33] was applied to perform the Spearman correlation analysis between each key gene and all remaining genes, generating corresponding correlation coefficients. These coefficients were subsequently ranked, and the clusterProfiler package (v 4.8.3) was employed to conduct GSEA using the ordered results. Significant enrichment was defined as |NES| > 1 and *p* < 0.05. Visualization of GSEA results was carried out using the GseaVis package (v 0.1.0) (H. [34]).

### 2.7. Immune Infiltration Analysis

To evaluate immune cell infiltration in PD, the CIBERSORT algorithm was applied to estimate the abundances of 22 immune cell types between the PD and control groups in the GSE10334 dataset [35]. The Wilcoxon test was subsequently performed to assess differences in infiltration levels of these 22 immune cell types between PD and control samples (*p* < 0.05), and violin plots were generated using the ggplot2 package (v 3.4.1) to visualize the results. Additionally, associations between key genes and differentially infiltrated immune cells across all samples in the GSE10334 dataset were analyzed using the Spearman correlation in the psych package (v 2.4.3). A threshold of |cor| > 0.3 and *p* < 0.05 was adopted, and correlation heat maps were constructed with the ggplot2 package (v 3.4.1).

### 2.8. GeneMANIA and Molecular Regulatory Network Analysis

To elucidate the functions of key genes, they were entered into the GeneMANIA database, and an interaction network with functionally similar genes was constructed to identify and characterize their roles.

To determine upstream regulators of the key genes, the TRRUST database was used to predict transcription factors (TFs) binding to these genes, and Cytoscape software (v 3.10.2) was applied to construct the TF-mRNA regulatory network.

To investigate upstream microRNAs (miRNAs) of the key genes in PD, two databases, TargetScan (http://www.targetscan.org) and miRWalk, were independently applied to predict mRNA–miRNA interaction pairs. The intersection of miRNAs predicted by both databases was used to identify key miRNAs. A miRNA–mRNA regulatory network was subsequently constructed with Cytoscape software (v 3.10.2) based on these key miRNAs and their target mRNAs.

### 2.9. Drug Prediction and Molecular Docking Analysis

To identify potential therapeutic agents for PD, the Comparative Toxicogenomics Database (CTD, https://ctdbase.org/) was applied to predict drugs or compounds associated with key genes. The Top 20 candidates were selected to construct a drug–key gene regulatory network using Cytoscape software (v 3.10.2). These selected compounds were defined as key drugs for molecular docking. Three-dimensional molecular structures of the drugs were retrieved from the PubChem database. Subsequently, key genes were queried in the UniProt and PDB databases to obtain the highest resolution receptor structures. Molecular docking simulations were carried out using CB-dock2, and the conformation with the lowest docking score was downloaded. PyMOL (v 2.6) was then employed to visualize docking conformations. A lower docking score (binding energy) indicated stronger docking efficacy, and binding energies below −5 kcal/mol were regarded as evidence of favorable affinity between drugs and key gene targets.

### 2.10. Molecular Dynamics (MD) Simulation

To further validate the results of molecular docking, we conducted MD simulations. Specifically, the GROMACS 2024.4 software [36] was used to perform a 20 ns MD simulation to further verify the rationality and reliability of the molecular docking results. The entire process followed the rules of the AMBER99SB-ILDN force field, and the TIP3P water model was used.

Before the experiment began, two stages of pre-equilibration treatments were carried out. In the first stage, the NVT system was used to simulate the equilibration at 300 K for 100 ps to stabilize the system temperature. In the second stage of equilibration, the NPT system was employed to conduct the simulation at 1 bar for 100 ps to stabilize the system pressure.

In the experiment, the parameters and topology files of the protein and small molecule ligand were generated by the “AMBER14SB” force field and the AMNER gaff force field, respectively. In order to avoid the complex long-range interactions caused by the periodic boundary conditions, the periodic boundary conditions were set and optimized, and the simulation system was filled with water molecules. Meanwhile, to make the simulation system electrically neutral, some solvent water molecules were replaced by Na^+^ and Cl^−^ with a concentration of 0.15 mol/L. In addition, the steepest descent method was used to minimize the energy consumption of the entire system.

During the simulation, we calculated the root mean square deviation (RMSD), root mean square fluctuation (RMSF), and energy to reveal the changes in the position of the conformation during the simulation, the flexibility, and motion intensity of the protein amino acids.

### 2.11. Real-Time Quantitative Polymerase Chain Reaction (RT-qPCR) Validation

To determine whether the expression of key genes in clinical samples corresponded with bioinformatic predictions, gingival tissue samples were collected from 10 healthy controls and 10 patients with PD recruited from the Second Affiliated Hospital of Guizhou University of Traditional Chinese Medicine. All participants signed and completed a form indicating their informed consent, and the ethical approval agency was the Ethics Committee of the Second Affiliated Hospital of Guizhou University of Traditional Chinese Medicine (Ethics Number: KY20241217). GraphPad Prism 10 was employed to generate plots and calculate *p* values. Total RNA was extracted from the 20 samples using the TRIzol method (Vazyme Biotech Co., Nanjing, China) and subsequently reverse transcribed into complementary DNA (cDNA) with the Hifair III 1st Strand cDNA Synthesis SuperMix for qPCR kit (Yysen Biotechnology, Shanghai, China). RT-qPCR was then performed using 2× Universal Blue SYBR Green qPCR Master Mix (Servicebio, Wuhan, China), with GAPDH serving as the internal reference for quantifying gene expression. Primer sequences are provided in Table 1. Expression levels of biomarkers between the two groups were calculated using the 2^−ΔΔCt^ algorithm and compared with the *t*-test (*p* < 0.05).

### 2.12. Immunohistochemistry

To evaluate protein-level expression of key genes in periodontal tissues, immunohistochemical (IHC) staining was conducted on paraffin-embedded sections from 10 PD patients and 10 healthy controls. Tissue sections were deparaffinized, rehydrated, and subjected to antigen retrieval with citrate buffer. Endogenous peroxidase activity was blocked using 3% hydrogen peroxide. After blocking with 5% bovine serum albumin, the sections were incubated overnight at 4°C with primary antibodies against ICAM1 (Cat# 10831-1-AP), CXCL12 (Cat# 17402-1-AP), and MMP3 (Cat# 17873-1-AP), all obtained from Wuhan Sanying Biotechnology Co., Ltd.

On the following day, sections were incubated with horseradish peroxidase-conjugated secondary antibodies, developed using 3,3⁣′-diaminobenzidine, and counterstained with hematoxylin. Stained slides were visualized under a light microscope, and marker expression was quantified with ImageJ software by calculating the integrated optical density (IOD). Statistical comparisons between groups were performed using the *t*-test, with *p* < 0.05 considered statistically significant.

### 2.13. Statistical Analysis

Bioinformatic data were analyzed using R software (v 4.3.1). The Wilcoxon test or *t*-test was applied to evaluate differences between the two sample groups. A *p* value < 0.05 was regarded as statistically significant.

## 3. Results

### 3.1. Identification of DEGs

**Figure 1:**
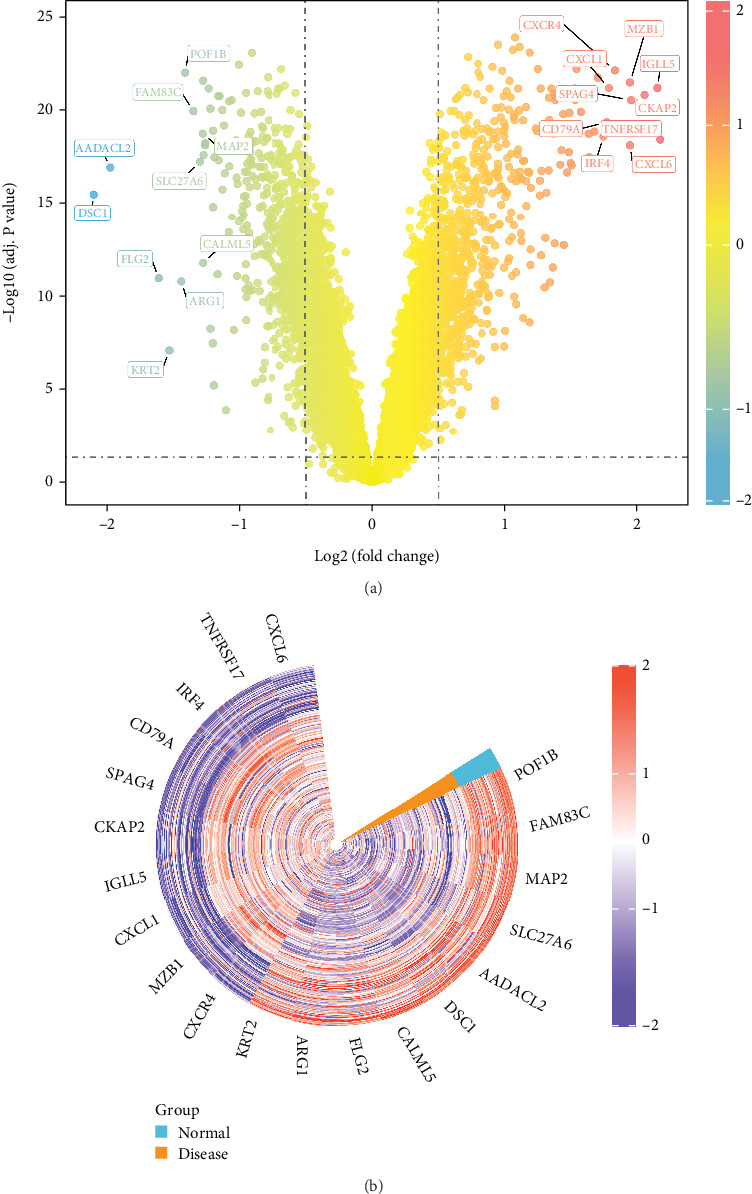
Identification of DEGs. (a) Volcano plot of DEGs between PD and control groups. (b) Heat map displaying DEG expression patterns.

Differential expression analysis of the GSE10334 dataset identified 1316 DEGs (*p* adjust < 0.05 and |log2FC| > 0.5). Among these, 657 genes were upregulated, and 659 genes were downregulated in PD samples compared with controls (Figure 1a,b) (Table S2).

### 3.2. Acquisition of Candidate Genes

**Figure 2:**
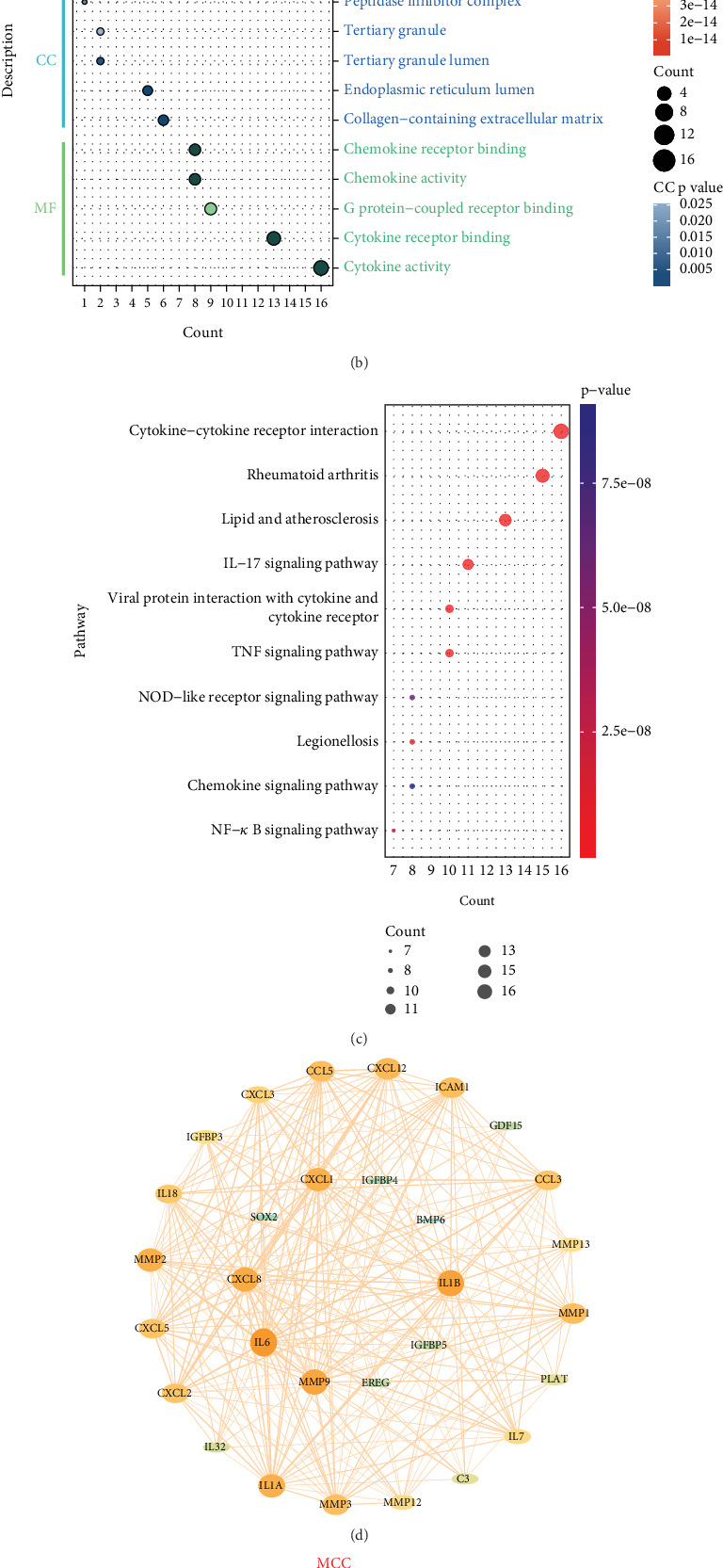
Acquisition of candidate genes. (a) Venn diagram of candidate gene selection. (b) GO enrichment results of intersection genes. (c) KEGG pathway enrichment of intersection genes. (d) PPI network of hub genes. (e) Intersection of the Top 15 proteins obtained using five CytoHubba algorithms.

The intersection of 1316 DEGs and 153 SASPRGs yielded 30 differentially expressed SASP-related genes in PD (candidate genes) for subsequent analysis (Figure 2a). GO enrichment analysis revealed that these 30 candidate genes were enriched in 945 GO signaling pathways (*p* < 0.05), including 903 BP pathways (e.g., leukocyte migration and myeloid leukocyte migration) (Table S3), 5 CC pathways (e.g., collagen-containing extracellular matrix and endoplasmic reticulum lumen) (Table S4), and 37 MF pathways (e.g., cytokine activity and cytokine receptor binding) (Table S5). Enriched biological functions were ranked in descending order by count, and the top five included leukocyte migration, collagen-containing extracellular matrix, and cytokine activity (Figure 2b). KEGG enrichment analysis demonstrated that these genes were associated with 53 pathways (*p* < 0.05) (Table S6). The Top 10 pathways, ranked by count, included cytokine–cytokine receptor interaction and rheumatoid arthritis (Figure 2c).

A PPI network comprising 30 nodes and 254 interactions was then constructed (confidence score ≥ 0.4), indicating relatively strong connectivity among these proteins. Representative interactions included CXCL1 with CXCL12 and MMP9 with MMP3 (Figure 2d).

The Top 15 proteins identified by MCC, MNC, DMNC, degree, and EPC algorithms were intersected, yielding 8 final candidate genes: MMP1, CCL3, ICAM1, CXCL12, CCL5, CXCL2, IL1A, and MMP3 (Figure 2e).

### 3.3. Identification of Key Genes

**Figure 3:**
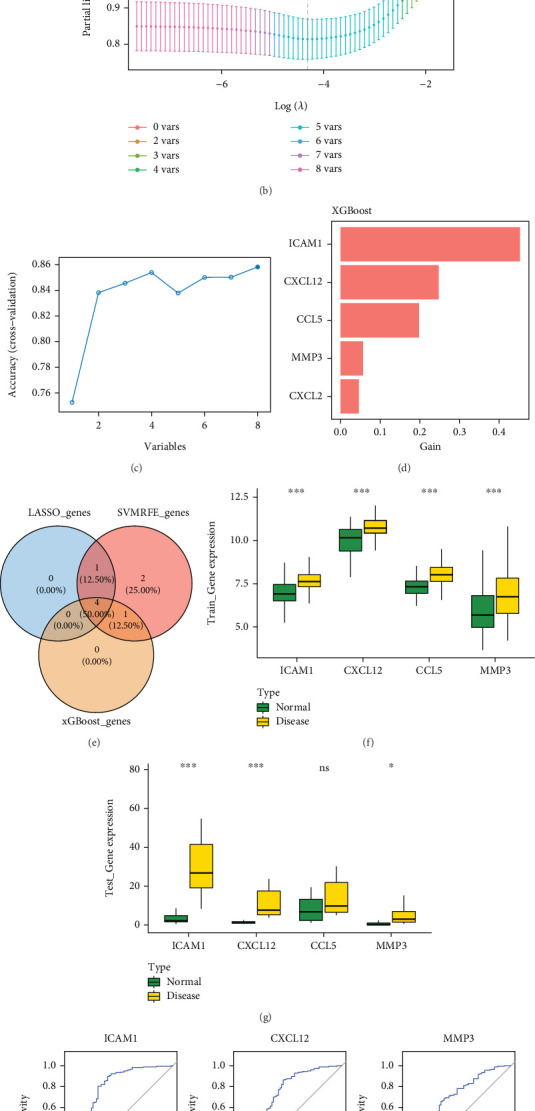
Identification of key genes. (a) LASSO regression coefficient path. (b) LASSO regression partial likelihood deviance. (c) SVM-RFE cross-validation results. (d) XGBoost analysis results. (e) Venn diagram of feature gene intersections from three machine learning methods. (f) Expression analysis of feature genes in the training dataset GSE10334. (g) Expression analysis of feature genes in the validation dataset GSE223924. (h) ROC analysis of candidate key genes in GSE10334. (i) ROC analysis of candidate key genes in GSE223924.

Using the LASSO algorithm, when log(lambda.min) = −4.3153, five genes were identified: ICAM1, CXCL12, CCL5, IL1A, and MMP3, designated as LASSO genes (Figure 3a,b). SVM-RFE analysis indicated the highest predictive accuracy when the optimal number of variables was eight, yielding ICAM1, CCL5, CXCL12, CCL3, IL1A, CXCL2, MMP3, and MMP1, defined as SVM-RFE genes (Figure 3c). Similarly, XGBoost analysis of all GSE10334 samples identified five high-importance genes, ICAM1, CXCL12, CCL5, MMP3, and CXCL2, referred to as XGBoost genes (Figure 3d).

The intersection of LASSO, SVM-RFE, and XGBoost results produced four overlapping genes: ICAM1, CXCL12, CCL5, and MMP3, denoted as characteristic genes (Figure 3e).

Expression of these characteristic genes was validated using the Wilcoxon test in both GSE10334 and GSE223924 datasets (*p* < 0.05). In the GSE10334 dataset, ICAM1, CXCL12, CCL5, and MMP3 were significantly upregulated in PD samples (Figure 3f). In the GSE223924 dataset, ICAM1, CXCL12, and MMP3 showed significant expression, with expression trends consistent with those observed in GSE10334 (Figure 3g). Therefore, ICAM1, CXCL12, and MMP3 were considered candidate key genes. ROC curve analysis was subsequently performed across all samples from GSE10334 and GSE223924. In GSE10334, AUC values for ICAM1 and CXCL12 exceeded 0.7, while MMP3 remained below this threshold (Figure 3h). In GSE223924, AUC values for ICAM1, CXCL12, and MMP3 all exceeded 0.7 (Figure 3i). Based on these results, ICAM1 and CXCL12 were identified as key genes for subsequent analyses (AUC > 0.7).

### 3.4. Construct of an ANN of Two Key Genes

**Figure 4:**
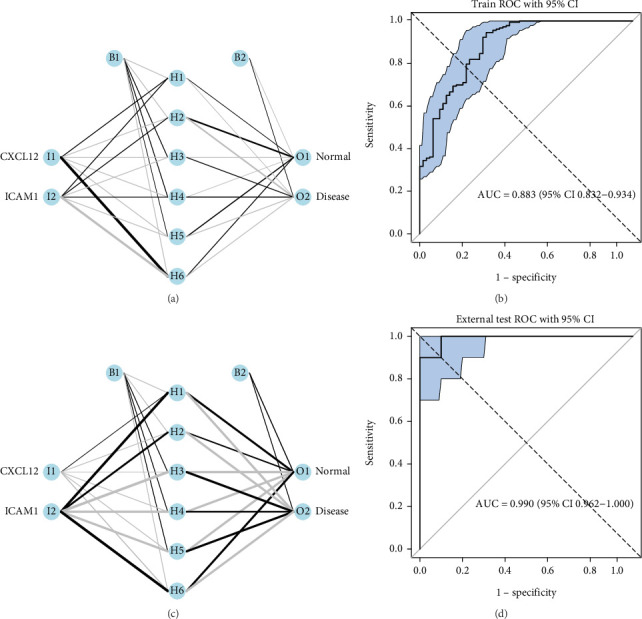
Construction of an artificial neural network for two key genes. ANN model of the training dataset: (a) ANN model structure and (b) ROC curve. ANN model of the validation dataset: (c) ANN model structure and (d) ROC curve.

To assess the clinical diagnostic value of key genes, an ANN model incorporating CXCL12 and ICAM1 was constructed using all samples from the GSE10334 dataset to predict PD incidence. The results indicated that CXCL12 and ICAM1 possessed strong diagnostic value for PD (Figure 4a). ROC curve analysis further demonstrated an AUC of 0.883 (95% CI = 0.832–0.943) (Figure 4b), reflecting relatively high predictive accuracy. Comparable results were also observed in the GSE223924 dataset (AUC = 0.990, 95% CI = 0.962–1.000) (Figure 4c,d).

### 3.5. Biological Pathways, the Immune Microenvironment of Two Key Genes

**Figure 5:**
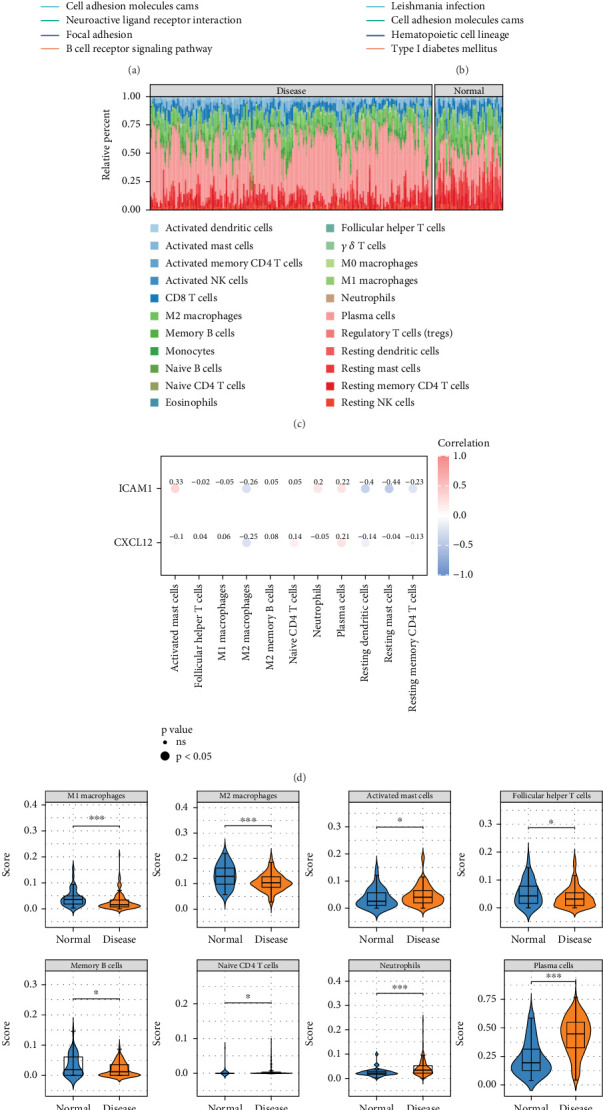
Biological pathways and immune microenvironment of two key genes. (a) GSEA enrichment results of CXCL12. (b) GSEA enrichment results of ICAM1. (c) Immune cell infiltration in training dataset samples. (d) Differential immune cell infiltration between the PD and control groups. (e) Correlations between differential immune cells and key genes.

GSEA revealed that CXCL12 was enriched in 75 pathways, including olfactory transduction (Figure 5a) (Table S7), while ICAM1 was enriched in 61 pathways, such as ribosome (|NES| > 1, *p* < 0.05) (Figure 5b) (Table S8). Moreover, both genes were jointly involved in *Leishmania* infection, cell adhesion molecules (CAMs), hematopoietic cell lineage, and Parkinson's disease pathways. These results suggest that these pathways may contribute to the pathogenesis of PD.

Immune infiltration analysis of 22 immune cell types in the GSE10334 dataset was conducted (Figure 5c). The Wilcoxon test revealed significant differences in 11 cell types, with activated mast cells, neutrophils, and plasma cells displaying higher infiltration levels in the PD group (*p* < 0.05) (Figure 5d). Additionally, ICAM1 expression showed a significant negative correlation with resting mast cells (cor = −0.44, *p* = 8.95e − 12) (Figure 5e) (Table S9).

### 3.6. GeneMANIA and the Regulatory Network of Two Key Genes

**Figure 6:**
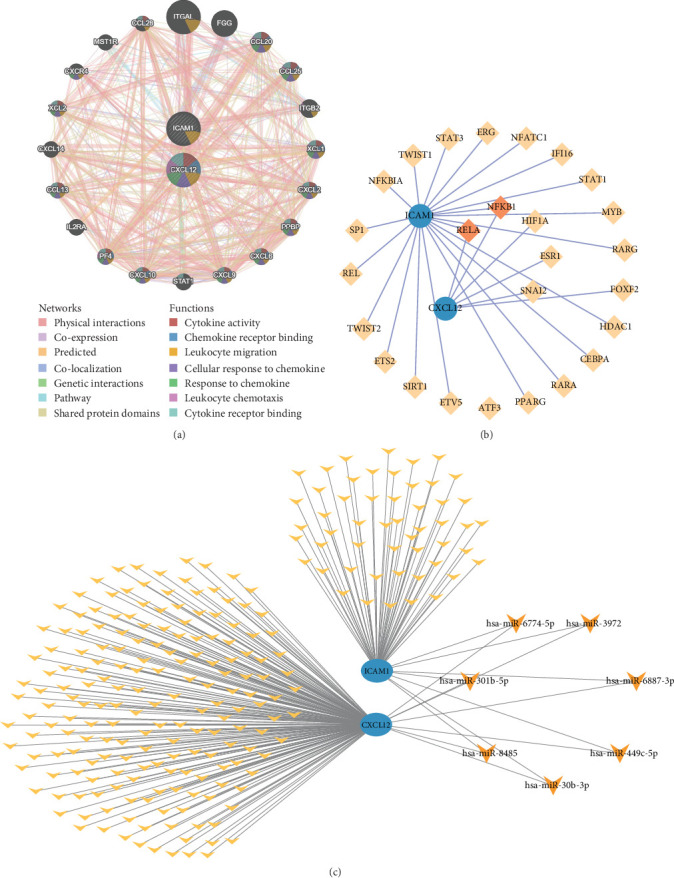
GeneMANIA and the regulatory networks of two key genes. (a) GeneMANIA analysis of key genes. (b) TF–mRNA regulatory network. (c) miRNA–mRNA regulatory network.

GeneMANIA analysis identified 20 genes functionally associated with CXCL12 and ICAM1, including ITGAL and FGG. These genes interacted through seven distinct modes of action, such as physical interactions and coexpression, and were closely related to seven biological functions, including cytokine activity and chemokine receptor binding. ICAM1 was primarily involved in leukocyte migration (Figure 6a).

A TF-mRNA regulatory network was constructed using the TF prediction database. This network revealed six TFs targeting CXCL12 and 21 targeting ICAM1 (Table S10). Among these, RELA and NFKB1 simultaneously targeted both genes (Figure 6b). Additionally, a miRNA–mRNA regulatory network was generated using Cytoscape software. CXCL12 was predicted to be targeted by 509 miRNAs in the TargetScan database and 984 miRNAs in the miRWalk database, with 190 overlapping miRNAs identified (Table S11). ICAM1 was predicted to be targeted by 628 miRNAs in TargetScan and 183 in miRWalk, with 59 overlapping miRNAs identified (Table S12). The miRNA–mRNA network further revealed that seven miRNAs, including hsa-miR-3972 and hsa-miR-301b-5p, simultaneously targeted both CXCL12 and ICAM1 (Figure 6c).

### 3.7. Drug Prediction of Two Key Genes

**Figure 7:**
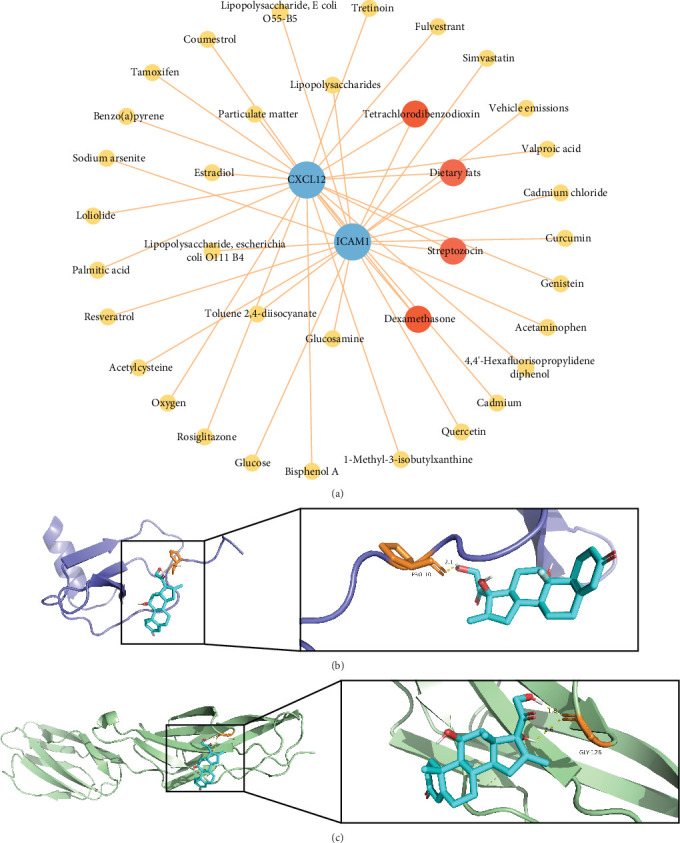
Drug prediction of two key genes. (a) Drug–gene regulatory network. (b) Molecular docking conformation of CXCL12 with dexamethasone. (c) Molecular docking conformation of ICAM1 with dexamethasone.

CTD analysis predicted 342 compounds or drugs targeting CXCL12 (Table S13), including tamoxifen and estradiol. A total of 774 compounds were predicted to target ICAM1, such as glucosamine and acetaminophen (Table S14). Among these, 174 compounds were predicted to simultaneously target both genes. Construction of a drug–key gene regulatory network using the Top 20 compounds revealed that streptozocin, dietary fats, dexamethasone, and tetrachlorodibenzodioxin targeted both CXCL12 and ICAM1 (Figure 7a). However, streptozocin is an antibiotic commonly used for diabetes modeling, dietary fats represent the collective oils from food sources, and tetrachlorodibenzodioxin is a highly toxic, persistent organic pollutant with no reported pharmacological application. Therefore, only dexamethasone was retained as the key compound for molecular docking. Docking analysis demonstrated that CXCL12 bound to dexamethasone with a binding energy of −6.2 kcal/mol, forming one hydrogen bond (bond length: 2.9 Å) (Figure 7b). ICAM1 bound to dexamethasone with a binding energy of −6.2 kcal/mol, forming two hydrogen bonds (bond lengths: 1.8 and 2.6 Å) (Figure 7c), suggesting a stable binding conformation with favorable affinity.

### 3.8. CXCL12 and ICAM1 Bound to Dexamethasone Relatively Stably

**Figure 8:**
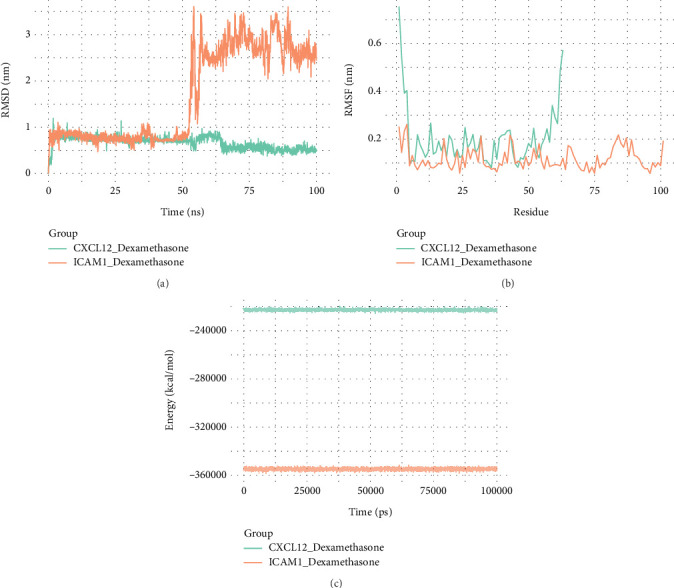
The results of molecular dynamics. (a) The RMSD of CXCL12 and ICAM1. The abscissa represents time, and the ordinate represents the RMSD value. (b) The RMSF of CXCL12 and ICAM1. The abscissa represents the atom serial number, and the ordinate represents the RMSF value. (c) The energy of CXCL12 and ICAM1 varying with time. The horizontal axis represents time, and the vertical axis represents the energy.

Additionally, the results of the MD simulation showed that the RMSD values of CXCL12 and ICAM1 were concentrated between 0.5 and 1, which suggested that their proteins were in a stable state from 1 to 50 ns and 5 to 100 ns (Figure 8a). Meanwhile, the RMSF values of CXCL12 and ICAM1 were concentrated in the ranges of 0.1–0.25 and 0.1–0.2, respectively; this indicated that the amino acid flexibility of these proteins was favorable and that their binding to the dexamethasone ligand was stable (Figure 8b). Furthermore, the energy values of CXCL12 and ICAM1 were low during the simulation and exhibited minimal fluctuations, which demonstrated that the binding of CXCL12 and ICAM1 to dexamethasone was relatively stable (Figure 8c).

### 3.9. High Expression of ICAM1, CXCL12, and MMP3 in PD Samples

**Figure 9:**
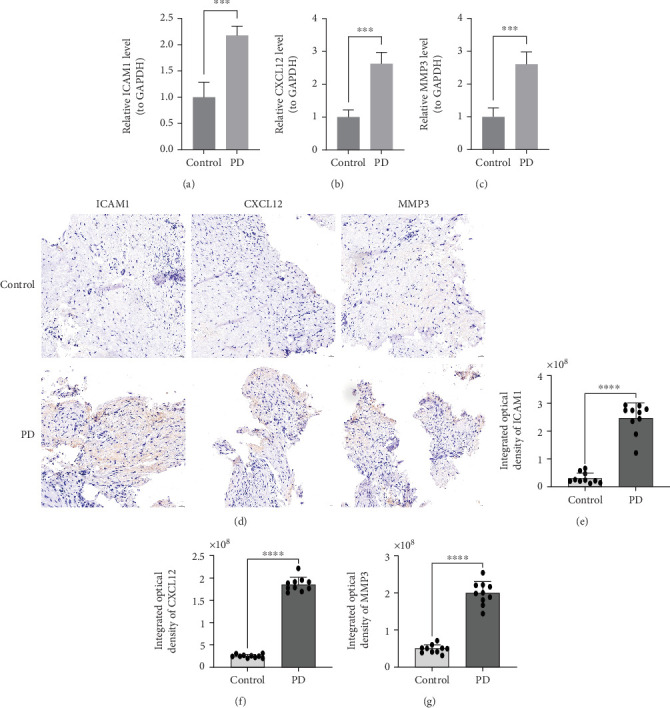
High expression of ICAM1, CXCL12, and MMP3 in PD samples. Expression of (a) ICAM1, (b) CXCL12, and (c) MMP3 in periodontal tissues. (d) IHC validation and quantification of ICAM1, CXCL12, and MMP3 expression in periodontal tissues. Quantification of IOD of (e) ICAM1, (f) CXCL12, and (g) MMP3 in control (*n* = 10) and PD (*n* = 10) groups. Control: healthy periodontal tissue (*n* = 10); PD: periodontitis tissue (*n* = 10).

Clinical samples were analyzed by RT-qPCR to validate the expression of ICAM1, CXCL12, and MMP3. The results confirmed that all three genes were significantly upregulated in PD samples (*p* < 0.001) (Figures 9a, 9b, and 9c), consistent with the expression profiles observed in transcriptome sequencing data. To further validate these findings at the protein level, IHC staining was performed on periodontal tissues from PD patients and healthy controls (*n* = 10 per group) using antibodies against ICAM1, CXCL12, and MMP3. As shown in Figure 9d, strong immunopositivity was observed for all three proteins in PD tissues compared with controls, with staining predominantly localized to epithelial and connective tissue regions of the gingiva.

Quantitative analysis of IOD further confirmed significantly elevated expression levels of ICAM1, CXCL12, and MMP3 in PD tissues relative to controls (*p* < 0.001) (Figures 9e, 9f, and 9g). These protein-level results were consistent with mRNA expression data from transcriptomic datasets and RT-qPCR validation, reinforcing the reliability of the findings. The upregulation of these SASP-related proteins in PD supports their potential role in mediating local inflammation and immune dysregulation.

## 4. Discussion

PD is a prevalent chronic inflammatory disease that often results in alveolar bone loss and tooth mobility [1]. As the disease advances, local inflammation intensifies, progressively impairing the structure and function of periodontal tissues. The SASP plays a pivotal role in diverse physiological and pathological processes, particularly in sustaining chronic inflammation. Previous studies have demonstrated that SASP contributes to the initiation and progression of PD (L. [7]). By releasing proinflammatory cytokines, chemokines, and matrix-remodeling enzymes, SASP reshapes the local microenvironment and amplifies inflammatory responses. In PD pathogenesis, SASP is regarded as a key factor driving local inflammatory cascades and tissue destruction (L. [7]). Moreover, studies have indicated that senescent cells, through bacterial interactions and secretion of SASP components, disrupt the local microenvironment and induce paracrine effects in adjacent cells. This process further aggravates chronic inflammation within periodontal tissues, ultimately accelerating alveolar bone loss [11]. Collectively, these findings highlight the central role of SASP in PD. Nevertheless, the precise mechanisms through which SASP modulates PD and the immune microenvironment remain insufficiently defined. In this study, bioinformatic approaches and transcriptomic analyses were applied to investigate SASP-related genes in PD, with particular focus on ICAM1 and CXCL12.

In this study, ICAM1 and CXCL12 were identified as key genes significantly upregulated in PD. ICAM1, an intercellular adhesion molecule belonging to the immunoglobulin superfamily [37], plays a pivotal role in inflammation and immune regulation. Previous studies have demonstrated that ICAM1 upregulation enhances leukocyte–endothelial adhesion, thereby facilitating immune cell infiltration and exacerbating local inflammation [38]. These findings confirmed elevated ICAM1 expression in PD samples, which is consistent with a role in modulating the immune microenvironment in PD [39]. Moreover, a significant negative correlation was observed between ICAM1 expression and resting mast cells (cor = −0.44, *p* = 8.95e − 12), indicating that ICAM1 may influence immune cell infiltration and serve as a potential regulator of immune responses in PD.

CXCL12 (stromal cell-derived factor 1) is another key gene implicated in PD. Acting through its receptor CXCR4, CXCL12 promotes immune cell migration and regulates local immune responses [40]. In this study, CXCL12 was found to be significantly upregulated in PD samples, suggesting its potential role in shaping the immune microenvironment of PD. GSEA further demonstrated that CXCL12 was enriched in multiple pathways, including *Leishmania* infection, CAMs, and hematopoietic cell lineages, which are closely associated with immune regulation, cell migration, and bone metabolism [41]. These findings indicate that CXCL12, through its participation in these pathways, may facilitate immune cell infiltration and intensify local inflammatory responses in PD [42].

The results of functional enrichment analysis are of particular significance, demonstrating that ICAM1 and CXCL12 are involved not only in classical inflammation-related pathways but also in additional biological mechanisms. Their coenrichment in CAMs and hematopoietic cell lineage pathways highlights their pivotal roles in immune cell migration and adhesion. Enrichment in the *Leishmania* infection pathway further indicates that these genes may aggravate chronic inflammation in PD by modulating interactions between immune cells and pathogens [41]. Moreover, the shared enrichment of ICAM1 and CXCL12 in Type 1 diabetes pathways suggests that these genes may exacerbate the onset and progression of PD in diabetic patients [43].

In the immune infiltration analysis, the immune microenvironment of PD patients exhibited significantly higher infiltration of activated mast cells, neutrophils, and plasma cells compared with healthy controls (X. [44]). These immune cell populations are integral to sustaining chronic inflammation in PD. Notably, the results of this study indicated that the expression level of ICAM1 was significantly negatively correlated with the infiltration abundance of resting mast cells. This negative correlation suggests that ICAM1 may lead to a decrease in the proportion of resting mast cells in local tissues by promoting their activation or recruitment. As an important CAM, one of the principal functions of ICAM1 is to mediate leukocyte adhesion to endothelial cells and subsequent transendothelial migration [45]. Although ICAM1 itself is not a direct activator of mast cells, the potent cell–cell interactions mediated by ICAM1 (e.g., adhesion to T cells and neutrophils) can create a highly inflammatory microenvironment [46, 47]. Within this environment, the concentrations of mast cell-activating factors, such as IL-33 and stem cell factor (SCF), are elevated, thereby driving the transition of resting mast cells to an activated state [48, 49]. Activated mast cells rapidly degranulate, releasing histamine, tryptase, and other inflammatory mediators, accompanied by changes in their cellular phenotype [50], which may result in a relative decrease in the proportion of cells labeled as “resting” within the overall cell population. Moreover, CXCL12 may amplify immune responses in PD by facilitating immune cell migration [42].

Overall, the findings of this study suggest potential roles of ICAM1 and CXCL12 in PD, indicating that these genes are key regulators of immune modulation and local inflammatory responses. Their diagnostic utility was further evaluated using an ANN model, which demonstrated high predictive accuracy. Future investigations should focus on elucidating the specific mechanisms of ICAM1 and CXCL12 in PD and assessing their potential clinical applications as therapeutic targets.

## 5. Limitation

Although ICAM1 and CXCL12 were successfully identified and validated in PD through bioinformatic analyses, RT-qPCR, and IHC, thereby providing insights into underlying molecular mechanisms, several limitations remain. First, the transcriptomic data obtained from public databases may contain noise or bias, and samples used for RT-qPCR and immunohistochemistry experiments were derived from a single center, and the sample size was relatively small, which may affect the generalizability of the results. Validation in larger, multicenter clinical cohorts is warranted. Second, although preliminary analyses of immune infiltration and gene-related mechanisms were conducted, the interactions among distinct immune cell types and their specific functions in PD require further experimental confirmation. Finally, although molecular docking predicted a strong binding affinity of dexamethasone with ICAM1 and CXCL12, this observation must be validated through animal studies and clinical trials to confirm its therapeutic efficacy.

## 6. Conclusion

In this study, ICAM1 and CXCL12 were identified and validated as key genes in PD through transcriptomic analyses, bioinformatic approaches, and machine learning methods. The findings indicate that these genes may serve important roles in immune modulation and local inflammatory responses in PD. Their diagnostic potential was further confirmed using an ANN model, which demonstrated high predictive accuracy. Future investigations should focus on elucidating the mechanisms of ICAM1 and CXCL12 and assessing their clinical utility in PD, thereby providing new avenues for early diagnosis and targeted therapeutic strategies.

## Figures and Tables

**Table 1:** Primer sequences used for RT-qPCR.

**Gene symbol**	**Primer**		**Sequence (5**⁣′**→3**⁣′**)**	**Product size (bp)**
NM_000201.3	ICAM1	F	ATGCCCAGACATCTGTGTCC	112
NM_000201.3	ICAM1	R	GGGGTCTCTATGCCCAACAA	112
NM_199168.4	CXCL12	F	CTACAGATGCCCATGCCGAT	226
NM_199168.4	CXCL12	R	GTGGGTCTAGCGGAAAGTCC	226
NM_002422.5	MMP3	F	TGAGGACACCAGCATGAACC	248
NM_002422.5	MMP3	R	ACTTCGGGATGCCAGGAAAG	248
NM_001256799.3	GAPDH	F	ATGGGCAGCCGTTAGGAAAG	135
NM_001256799.3	GAPDH	R	AGGAAAAGCATCACCCGGAG	135

Abbreviation: Bp, base pairs.

## Data Availability

The data that support the findings of this study are available from the corresponding author upon reasonable request.

## Supporting Information

Supporting Information 1 Table S1. SASP-related genes.

Supporting Information 2 Table S2. DEGs associated with PD from the GSE10334 dataset.

Supporting Information 3 Table S3. GO BP enrichment analysis of 30 SASP-related candidate genes.

Supporting Information 4 Table S4. GO CC enrichment analysis of 30 SASP-related candidate genes.

Supporting Information 5 Table S5. GO MF enrichment analysis of 30 SASP-related candidate genes.

Supporting Information 6 Table S6. KEGG pathway enrichment analysis of 30 SASP-related candidate genes.

Supporting Information 7 Table S7. GSEA enrichment results of CXCL12 in the GSE10334 dataset.

Supporting Information 8 Table S8. GSEA enrichment results of ICAM1 in the GSE10334 dataset.

Supporting Information 9 Table S9. Correlations between SASP-related gene expression and immune cell infiltration in the GSE10334 dataset.

Supporting Information 10 Table S10. Predicted TFs regulating CXCL12 and ICAM1 from the TF prediction database.

Supporting Information 11 Table S11. miRNAs targeting CXCL12 predicted by both TargetScan and miRWalk and their intersection.

Supporting Information 12 Table S12. miRNAs targeting ICAM1 predicted by both TargetScan and miRWalk and their intersection.

Supporting Information 13 Table S13. Compounds and drugs predicted to target CXCL12 using the CTD.

Supporting Information 14 Table S14. Compounds and drugs predicted to target ICAM1 using the CTD.

## References

[1] Sanz M., Del Castillo A. M., Jepsen S. (2020). Periodontitis and Cardiovascular Diseases. Consensus Report. *Journal of Clinical Periodontology*.

[2] Alghamdi B., Jeon H. H., Ni J. (2023). Osteoimmunology in Periodontitis and Orthodontic Tooth Movement. *Current Osteoporosis Reports*.

[3] Gasmi A., Gasmi Benahmed A., Noor S., Mujawdiya P. (2022). *Porphyromonas Gingivalis* in the Development of Periodontitis: Impact on Dysbiosis and Inflammation. *Archives of Razi Institute*.

[4] Santonocito S., Polizzi A. (2022). Oral Microbiota Changes During Orthodontic Treatment. *Frontiers in Bioscience-Elite*.

[5] Sälzer S., Graetz C., Dörfer C. E., Slot D. E., Van Der Weijden F. A. (2020). Contemporary Practices for mechanical Oral Hygiene to Prevent Periodontal Disease. *Periodontology 2000*.

[6] Santonocito S., Polizzi A., Marchetti E. (2022). Impact of Periodontitis on Glycemic Control and Metabolic Status in Diabetes Patients: Current Knowledge on Early Disease Markers and Therapeutic Perspectives. *Mediators of Inflammation*.

[7] Zhang L., Pitcher L. E., Yousefzadeh M. J., Niedernhofer L. J., Robbins P. D., Zhu Y. (2022). Cellular Senescence: A Key Therapeutic Target in Aging and Diseases. *Journal of Clinical Investigation*.

[8] Li X., Li C., Zhang W., Wang Y., Qian P., Huang H. (2023). Inflammation and Aging: Signaling Pathways and Intervention Therapies. *Signal Transduction and Targeted Therapy*.

[9] Kandhaya-Pillai R., Yang X., Tchkonia T., Martin G. M., Kirkland J. L., Oshima J. (2022). TNF‐*α*/IFN‐*γ* Synergy Amplifies Senescence-Associated Inflammation andSARS‐CoV‐2 Receptor Expression via hyper‐activatedJAK/STAT1. *Aging Cell*.

[10] Wang Q., Nie L., Zhao P. (2021). Diabetes Fuels Periodontal Lesions via GLUT1-Driven Macrophage Inflammaging. *International Journal of Oral Science*.

[11] Chen S., Zhou D., Liu O., Chen H., Wang Y., Zhou Y. (2022). Cellular Senescence and Periodontitis: Mechanisms and Therapeutics. *Biology*.

[12] Guo S., Fu L., Yin C. (2024). ROS-Induced Gingival Fibroblast Senescence: Implications in Exacerbating Inflammatory Responses in Periodontal Disease. *Inflammation*.

[13] Kang J., Zhan J., Yuan Y. (2025). CSF1R Inhibitor (PLX3397) Alleviates Experimental Periodontitis by Reducing Macrophage Senescence Through the PI3K/AKT/FOXO1 Signaling Pathway. *Scientific Reports*.

[14] Li S., Wen C., Bai X., Yang D. (2024). Association Between Biological Aging and Periodontitis Using NHANES 2009–2014 and Mendelian Randomization. *Scientific Reports*.

[15] Wang H., Wei R., Deng T., Zhang J., Shen Z. (2023). Identifying Immuno-Related Diagnostic Genes and Immune Infiltration Signatures for Periodontitis and Alopecia Areata. *International Immunopharmacology*.

[16] Yadalam P. K., Sharma S., Natarajan P. M., Ardila C. M. (2024). Gradient Boosting-Based Classification of Interactome Hub Genes in Periimplantitis With Periodontitis – An Integrated Bioinformatic Approach. *Frontiers in Oral Health*.

[17] Lee M. K., Woo S. R., Noh J. K. (2024). Prognostic Significance of SASP-Related Gene Signature of Radiation Therapy in Head and Neck Squamous Cell Carcinoma. *Molecular Cancer Therapeutics*.

[18] Saul D., Kosinsky R. L., Atkinson E. J. (2022). A New Gene Set Identifies Senescent Cells and Predicts Senescence-Associated Pathways Across Tissues. *Nature Communications*.

[19] Ritchie M. E., Phipson B., Wu D. (2015). Limma Powers Differential Expression Analyses for RNA-Sequencing and Microarray Studies. *Nucleic Acids Research*.

[20] Liu D.-B., He Y.-F., Chen G.-J. (2022). Construction of a circRNA-Mediated ceRNA Network Reveals Novel Biomarkers for Aortic Dissection. *International Journal of General Medicine*.

[21] Ito K., Murphy D. (2013). Application of *ggplot2* to Pharmacometric Graphics. *CPT: Pharmacometrics & Systems Pharmacology*.

[22] Zheng Y., Gao W., Zhang Q. (2022). Ferroptosis and Autophagy-Related Genes in the Pathogenesis of Ischemic Cardiomyopathy. *Frontiers in Cardiovascular Medicine*.

[23] Wu T., Hu E., Xu S. (2021). clusterProfiler 4.0: A Universal Enrichment Tool for Interpreting Omics *data*. *Innovation*.

[24] Pan S., Hu B., Sun J. (2022). Identification of Cross-Talk Pathways and Ferroptosis-Related Genes in Periodontitis and Type 2 Diabetes Mellitus by Bioinformatics Analysis and Experimental Validation. *Frontiers in Immunology*.

[25] Liu P., Xu H., Shi Y., Deng L., Chen X. (2020). Potential Molecular Mechanisms of Plantain in the Treatment of Gout and Hyperuricemia Based on Network Pharmacology. *Evidence-based Complementary and Alternative Medicine*.

[26] Li Y., Lu F., Yin Y. (2022). Applying Logistic LASSO Regression for the Diagnosis of Atypical Crohn’s Disease. *Scientific Reports*.

[27] Zhang Z., Zhao Y., Canes A., Steinberg D., Lyashevska O., written on behalf of AME Big-Data Clinical Trial Collaborative Group (2019). Predictive Analytics With Gradient Boosting in Clinical Medicine. *Annals of Translational Medicine*.

[28] Guan C., Ma F., Chang S., Zhang J. (2023). Interpretable Machine Learning Models for Predicting Venous Thromboembolism in the Intensive Care Unit: An Analysis Based on Data From 207 Centers. *Critical Care*.

[29] He Y., You G., Zhou Y. (2025). Integrative Machine Learning of Glioma and Coronary Artery Disease Reveals Key Tumour Immunological Links. *Journal of Cellular and Molecular Medicine*.

[30] Guo Q., Zhong X., Dang Z., Zhang B., Yang Z. (2025). Identification of GBN5 as a Molecular Biomarker of Pan-Cancer Species by Integrated Multi-Omics Analysis. *Discover Oncology*.

[31] Robin X., Turck N., Hainard A. (2011). pROC: An Open-Source Package for R and S+ to Analyze and Compare ROC Curves. *BMC Bioinformatics*.

[32] Wu Y., Xiao Q., Wang S., Xu H., Fang Y. (2023). Establishment and Analysis of an Artificial Neural Network Model for Early Detection of Polycystic Ovary Syndrome Using Machine Learning Techniques. *Journal of Inflammation Research*.

[33] Saidmamatov O., Jammatov J., Sousa C., Barros R., Vasconcelos O., Rodrigues P. (2023). Translation and Adaptation of the Adult Developmental Coordination Disorder/Dyspraxia Checklist (ADC) Into Asian Uzbekistan. *Sports*.

[34] Lu H., Xu Z., Shao L., Li P., Xia Y. (2024). High Infiltration of Immune Cells With Lower Immune Activity Mediated the Heterogeneity of Gastric Adenocarcinoma and Promoted Metastasis. *Heliyon*.

[35] Meng Z., Chen Y., Wu W. (2022). Exploring the Immune Infiltration Landscape and M2 Macrophage-Related Biomarkers of Proliferative Diabetic Retinopathy. *Frontiers in Endocrinology*.

[36] Gorelov S., Titov A., Tolicheva O., Konevega A., Shvetsov A. (2024). Determination of Hydrogen Bonds in GROMACS: A New Implementation to Overcome Memory Limitation. *Journal of Chemical Information and Modeling*.

[37] Tinajero-Rodríguez J. M., Ramírez-Vidal L., Becerril-Rico J. (2024). ICAM1 (CD54) Contributes to the Metastatic Capacity of Gastric Cancer Stem Cells. *International Journal of Molecular Sciences*.

[38] Zhou Q., Xu J., Xu Y., Sun S., Chen J. (2023). Role of ICAM1 in Tumor Immunity and Prognosis of Triple-Negative Breast Cancer. *Frontiers in Immunology*.

[39] Lv X., Luo C., Wu J. (2024). Integration of Single-Cell RNA Sequencing of Endothelial Cells and Proteomics to Unravel the Role of ICAM1 – PTGS2 Communication in Apical Periodontitis: A Laboratory Investigation. *International Endodontic Journal*.

[40] Cao Y., Ni Q., Bao C. (2024). The Role of Pericyte Migration and Osteogenesis in Periodontitis. *Journal of Dental Research*.

[41] Hiyoshi T., Domon H., Maekawa T. (2022). Neutrophil Elastase Aggravates Periodontitis by Disrupting Gingival Epithelial Barrier via Cleaving Cell Adhesion Molecules. *Scientific Reports*.

[42] Cambier S., Gouwy M., Proost P. (2023). The Chemokines CXCL8 and CXCL12: Molecular and Functional Properties, Role in Disease and Efforts Towards Pharmacological Intervention. *Cellular & Molecular Immunology*.

[43] Seoane-Collazo P., Rial-Pensado E., Estévez-Salguero Á. (2022). Activation of Hypothalamic AMP-Activated Protein Kinase Ameliorates Metabolic Complications of Experimental Arthritis. *Arthritis and Rheumatology*.

[44] Lu X., Liu J., Wei T., Zhou X. (2021). Elevated Salivary Activity of Mast Cell Chymase of Periodontitis Patients, and a New Bradykinin Generation Cascade, Mediating the Cross-Talks Between Mast Cell and Gingival Fibroblast. *International Immunopharmacology*.

[45] Haydinger C. D., Ashander L. M., Tan A. C. R., Smith J. R. (2023). Intercellular Adhesion Molecule 1: More Than a Leukocyte Adhesion Molecule. *Biology*.

[46] Fan K.-Q., Huang T., Yu J.-S., Li Y.-Y., Jin J. (2024). The Clinical Features and Potential Mechanisms of Cognitive Disorders in Peripheral Autoimmune and Inflammatory Diseases. *Fundamental Research*.

[47] Tsang C.-M., Wong C.-K., Ip W.-K., Lam C. W.-K. (2005). Synergistic Effect of SCF and TNF-Alpha on the Up-Regulation of Cell-Surface Expression of ICAM-1 on Human Leukemic Mast Cell Line (HMC)-1 Cells. *Journal of Leukocyte Biology*.

[48] Huang B., Lei Z., Zhang G.-M. (2008). SCF-Mediated Mast Cell Infiltration and Activation Exacerbate the Inflammation and Immunosuppression in Tumor Microenvironment. *Blood*.

[49] Molfetta R., Lecce M., Milito N. D. (2023). SCF and IL-33 Regulate Mouse Mast Cell Phenotypic and Functional Plasticity Supporting a Pro-Inflammatory Microenvironment. *Cell Death & Disease*.

[50] Marshall J. S., Portales-Cervantes L., Leong E. (2019). Mast Cell Responses to Viruses and Pathogen Products. *International Journal of Molecular Sciences*.

